# Effects of graded levels of *Azadirachta indica* seed oil on growth performance and biochemical profiles of broiler chickens

**DOI:** 10.1002/vms3.162

**Published:** 2019-03-03

**Authors:** Vanessa Mafouo Sonhafouo, Jean Raphaël Kana, Kissel Nguepi Dongmo

**Affiliations:** ^1^ Animal Nutrition and Production Research Unit Department of Animal Production Faculty of Agronomy and Agricultural Sciences Dschang Cameroon

**Keywords:** *Azadirachta indica*, biochemical indices, broiler chicken, growth performance, seeds oil

## Abstract

Because of speculated risk in generating antibiotic resistance in pathogenic microbiota, natural products from plant origin due to their diverse biological activities, have recently gained a great attention in animal nutrition. This study was designed to evaluate graded levels of neem seed oil on growth performance of broiler chickens. A total of 400‐day‐old chicks were randomly allocated to five experimental treatment groups. Experimental rations consisted of supplementing basal diet (*R*0^−^) with 1 g antibiotic (*R*0 + ), 15, 20 and 25 g neem seed oil/kg of feed. Data were recorded on feed intake, weight gain, feed conversion ratio, and serum biochemical parameters. Result revealed that feeding broiler chicks with 25 g of neem oil/kg of feed resulted in a marked (*P* < 0.05) decreased in feed intake as compared to the other treatments in the starter phase. The average live body weight and the weight gain decreased with increasing level of neem oil in the ration. Supplementation of poultry feed with graded levels of neem oil has no marked (*P* > 0.05) effect on carcass yield and relative weight of organs except for liver weight which significantly (*P* < 0.05) increased with the highest dose of neem oil. Serum content in total proteins, total cholesterol, HDL‐cholesterol, aspartate aminotransferase (AST), alanine aminotransferase (ALT) and creatinine were not significantly (*P* > 0.05) affected by the graded levels of neem oil. LDL‐cholesterol significantly (*P* < 0.05) decreased with diets supplemented 20 g of neem oil/kg while triglycerides significantly increased with the highest doses of oil (20 and 25 g/kg) as compared to the negative and positive control rations. In conclusion, feeding broilers with *Azadirachta indica* seed oil has no beneficial effect on growth performance but may lead to the production of low‐cholesterol chicken meat as demand by health‐conscious consumers.

## Introduction

Sub therapeutic use of antibiotics in the poultry industry for decades helped to maintain the equilibrium of the gut ecosystem and improve growth performance of chickens through good nutrition (Huyghebaert *et al*. 2011). However, this practice although very effective, has been questioned, given the accumulation of antibiotic residues in livestock products and the resistance developed by pathogenous microbes in poultry farms (Kabir 2009). As consequence, farmers are more and more fighting against fatal diseases in poultry through the use of active compounds of plant origin. Plant products such as essential oils, spices, plant extracts and oil such as neem seed oil were investigated as a possible solution that would address public health concerns without compromising the efficiency of poultry production. However, there is no single product that has been successful in replicating the consistent effects of antibiotic growth promoters.

Neem *(Azadirachta indica)*, is a tropical plant widely distributed in Africa and available all year round (Koona & Budida 2011; Ogbuewu *et al*. 2011). This plant is well adapted to the climatic and edaphic conditions of the rainforest and Sahelian tropical forest zones. Its leaves have a very bitter taste and have a garlic smell. Recent studies on the effects of neem leaves on broiler and table egg production have been documented (Bonsu *et al*. 2012; Jahanzeb *et al*. 2012). Those studies have shown that neem leaves at 2.5 g/kg feed have very beneficial effects on growth performance and hemato‐biochemical parameters in broilers (Jahanzeb *et al*. 2012). Ogbuewu *et al*. (2010, 2011) investigated the effect of dietary *A*. *indica* oil on body weight gain, linear body measurements and blood chemistry of pre‐puberal rabbits. The result revealed that rabbits could tolerate up to 15% dietary inclusion of neem oil without deleterious effects on body weight gain, linear body measurements, and reproductive tract morphometry and biochemical parameters. *A*. *indica* seed oil contains triterpenoid compounds such as azadirachtin, gedunine, nimbine and nimbidine which have antibacterial and antifungal properties (Makeri *et al*. 2007; Valarmathy *et al*. 2010) that can make its use as an alternative to antibiotic. These compounds in poultry feed can produce additive or synergistic effects on production performance (Valarmathy *et al*. 2010). The objective of the present study was to determine the effect of *Azadirachta indica* seeds oil as antibiotic substitute on the growth performance, carcass characteristics and biochemical profile of broiler chickens.

## Materials and methods

### Study area

This study was carried out at the Poultry Unit of the Teaching and Research Farm of the Faculty of Agronomy and Agricultural Sciences of the University of Dschang, Cameroon. Dschang, is located in the West region of Cameroon at 05° 26′ N and 10° 26′ E. The area experiences a wet season from March to November and a hot dry season for the rest of the year. Maximum ambient temperature is around 21°C, while an average annual rainfall of 2000 mm is prevalent.

### Experimental birds

A total of 400‐day‐old Cobb 500 strain chicks with an average body weight of 38 g obtained from a local hatchery were randomly divided into 25 floor pens (each 10 × 15 feet) of 16 chicks each and five replicates per treatment group. Birds were provided with *ad libitum* access to feed and water during the experiment period which lasted for 49 days. Birds were given vaccines in drinking water against Newcastel disease and Infectious Bronchitis on the 8th day with a booster dose on the 23th day of age, and against Gumboro disease on the 10th day of age. Coccidiosis prevention was done using Vetacox^®^ for 3 consecutive days per week from the 2nd to the 6th week of age. Birds were administered commercial antistress (Amintotal^®^, LAPROVET, Tours Cedex 2, France) in drinking water during the first 3 days upon arrival, after each vaccination, weighing session and transfer from brooding to finishing building.

### Experimental diet

Fresh neem fruits were collected at Garoua in the Northern Region of Cameroon between September and October 2017. They were thoroughly washed with running tap water and separated from the sheet manually. The seeds were oven dried at 50°C until constant moisture content was achieved. The dried seeds were ground in a mill for size reduction. The extraction of oil was carried out by kneading the paste. The phytochemical analysis of the extracted oil sample was carried out by Chromatographic method as described by Talukdar *et al*. (2010). The analysis revealed that alkaloids, flavonoids, terpenoids, phenols and steroids were present (Table 1).

**Table 1:** Phytochemical composition of neem oil

Contents	Components	Quantity
Sterols	Cholesterol	0.12 mg/kg
	Campesterol	0.34 mg/kg
	Campestanol	0.01 mg/kg
	Stigmasterol	0.50 mg/kg
	*β*‐Sitosterol	2.70 mg/kg
	Stigmastanol	0.05 mg/kg
	Avenasterol	0.17 mg/kg
Triterpenoids	Azadirachtine A	3.46 mg/kg
	Azadirachtine B	0.55 mg/kg
Fatty acids	Stearic acid	16.93%
	Oleic acid	43.68%
	Petroselenic acid	0.45%
	Linoleic acid	19.78%
	Linolenic acid	0.45%

Experimental diet calculated based on NRC (1994) tables of feed composition consisted of control ration without any supplement (*R*_0_^−^), positive control supplemented with 1 g of antibiotic/kg of feed (*R*_0_^+^) and three others rations supplemented with 15 g (*R*_15_), 20 g (*R*_20_) and 25 g (*R*_25_) of neem oil/kg of feed. The antibiotic used in positive control ration was Doxycycline^®^. The proximate composition of the basal diet is summarized in Table 2.

**Table 2:** Composition of the basal diet

Ingredients (%) starter		Finisher
Maize	59	65
Wheat bran	5	5
Cotton seed cake (50%)	6	4
Soybean meal (49%)	20	15
Fish meal (60%)	4	5
Shellfish	0.75	1
Bone meal	0.25	0
Premix 5%*	5	5
Total	100	100
Chemical composition (calculated)		
Metabolizable energy (kcal/kg)	2951.91	3006.85
Crude protein (%)	22.53	20.38
Energy/protein	130.99	147.54
Lysine (%)	1.32	1.19
Methionine (%)	0.46	0.45
Calcium (%)	1.07	1.15
Non‐phytate phosphorus (%)	0.5	0.48
Calcium/phosphore	2.16	2.41
Crude fibres (%)	4.96	4.94
Price/kg (FCFA, 550 FCFA≈1 USD)	273.59	147.54

* Premix: Mineral Nitrogen Mineral Complex: PB = 40%, Calcium = 8%, Phosphorus = 2.05%, Lysine = 3.3%, Methionine = 2.40%, ME = 2078 Kcal/kg, Vit A: 3 000 000 UI, Vit D 3: 600 000 UI, Vit E: 4000 mg, Vit K: 500 mg, Vit B1: 200 mg, VitB2: 1000 mg, Vit B6: 400 mg, Vit B12: 4 mg, Iron: 8000 mg, Cu: 2000 mg, Zn : 10 000 mg, Se: 20 mg, Mn: 14 000 mg, CP = crude protein, ME = Metabolizable energy, FCFA = Franc CFA (1 USD≈ 550 FCFA).

### Parameters measured

Pen body weights were recorded at 7, 14, 21, 28, 35, 42 and 49 day of age. Feed intake was determined for the same time period. At 49 days of age, 10 birds per treatment were randomly selected, fasted for 24 h weighed, slaughtered and eviscerated to evaluate and record carcass yield as preceded by Kana *et al*. (2017). Dressing percentage was calculated by dividing the carcass weight by live body weight of the bird and the result was expressed as a percentage. The length of the intestine was measured with the cut done from the start of the duodenal loop to the end of the cloacal. The density of the intestine was calculated by dividing the intestine weight by its length (Kana *et al*. 2017).

Blood samples of slaughtered birds were collected in non‐heparinised tube and serum was collected for the determination of AST, ALT, creatinine, urea, total proteins, total cholesterol, HDL‐cholesterol and LDL‐cholesterol. The AST and ALT content were estimated using the nicotinamide adenine dinucleotide dehydrogenase (NADH) oxidation reaction method. The serum (0.1 mL) was added to 1.0 mL of auto‐reagent and incubated at 37°C for 3 min. The absorbance was measured at 340 nm, and the values were expressed as unit L^−1^. Serum creatinine and urea levels were evaluated by colorimetric method as described by Bergmeyer & Wanlefeld (1980) and the results were expressed as mg/dL. Total serum protein content was determined by the Biuret method and serum cholesterol by the enzymatic colorimetric method.

### Statistical analysis

Results recorded on growth and serological parameters were subjected to one‐way analysis of variance procedure using the Statistical Package for Social Sciences (SPSS 20.0) computer software. A *P*‐value of <0.05 was considered a significant difference among groups and the comparison of means was made using Duncan's Multiple Range Test (Steel & Torrie 1984).

## Results

### Phytochemical content of neem oil

Phytochemical analysis (HPLC‐MS and GC‐MS) of neem oil identified 41 compounds with about 39.02% fatty acids, 36.58% triterpenoids and 24.40% sterols. The major fatty acids are oleic acid (43.68%), linoleic acid (19.78%) and stearic acid (16.93%). While the most prominent triterpenoids are Azadirachtin A (3.46 mg/kg) and Azadirachtin B (0.55 mg/kg) (Table 1). Although present in very small amounts, the major sterols are *β*‐Sitosterol (2.70 mg/kg) and Stigmasterol (0.50 mg/kg).

### Growth performance

The effects of graded levels of neem oil on feed intake, live body weight, weight gain and feed conversion ratio are summarized in Table 3. Feed intake decreased with increasing level of oil during the starter phase (days 1–21), meanwhile during the finisher phase (days 22–49) and throughout the entire period of the study (days 1–49), there was no significant (*P* > 0.05) effect of neem seed oil on feed intake. Feeding broilers with graded levels of neem oil had no marked effect on weight gain and feed conversion ratio during the starter phase. During the finisher phase and the entire period of the study, when compared to the negative control ration, feeding broilers with graded levels of neem oil has no significant (*P* > 0.05) effect on live body weight, weight gain and feed conversion ratio (FCR). However, when compared to the positive control ration supplemented with antibiotic, body weight gain markedly (*P* < 0.05) reduced with neem oil whatever the dose in the ration.

**Table 3:** Growth performance of broiler chickens as affected by graded levels of neem seed oil

Study period (Days)	Controls		Treatments			*P‐*value
	*R*_0_−	*R*_0_+	*R*_15_	*R*_20_	*R*_25_	
Feed intake (g)						
01–21	942.66 ± 21.39^ab^	921.35 ± 34.73^ab^	956.46 ± 26.14^a^	906.51 ± 31.08^b^	900.33 ± 33.42^b^	0.035
22–49	3343.64 ± 84.20	3250.83 ± 50.44	3303.95 ± 103.59	3372.85 ± 98.47	3357.50 ± 74.93	0.192
01–49	4286.30 ± 100.04	4172.18 ± 54.72	4260.41 ± 91.60	4279.37 ± 101.42	4257.82 ± 93.08	0.303
Live body weight (g)						
01–21	520.05 ± 41.21^ab^	555.76 ± 42.92^b^	511.64 ± 33.73^ab^	528.40 ± 47.32^ab^	474.51 ± 22.51^b^	0.047
22–49	1919.74 ± 294.54^b^	2335.37 ± 160.31^a^	1970.00 ± 193.27^b^	1957.94 ± 233.99^b^	1759.65 ± 255.77^b^	0.013
Weight gain (g)						
01–21	481.52 ± 41.21	517.23 ± 42.92	507.22 ± 35.98	525.10 ± 50.48	467.62 ± 24.01	0.157
22–49	1389.31 ± 293.96^b^	1766.48 ± 168.17^a^	1379.49 ± 187.62^b^	1337.57 ± 238.86^b^	1207.93 ± 231.38^b^	0.013
01–49	1870.83 ± 304.37^b^	2283.71 ± 153.28^a^	1886.70 ± 181.55^b^	1862.66 ± 224.77^b^	1675.55 ± 234.35^b^	0.007
Feed conversion ratio						
01–21	1.97 ± 0.17	1.79 ± 0.17	1.89 ± 0.16	1.74 ± 0.18	1.93 ± 0.15	0.204
22–49	2.51 ± 0.60^ab^	1.86 ± 0.21^b^	2.45 ± .48^ab^	2.60 ± 0.58^a^	2.87 ± 0.60^a^	0.066
01–49	2.35 ± 0.43^a^	1.83 ± 0.14^b^	2.28 ± 0.29^a^	2.33 ± 0.30^a^	2.58 ± 0.38^a^	0.024

^a,b^Means with different superscript for the same parameters in each row are significantly different (*P* < 0.05). *R*_0_−: 0 g of neem oil/kg, *R*_0_+: *R*_0_− + 1 g of Doxycicline/kg, *R*_15_: *R*_0_− + 15 g of neem oil/kg, *R*_20_: *R*_0_− + 20 g neem oil/kg, *R*_25_: *R*_0_− + 25 g of neem oil/kg, *P*: Probability.

### Carcass characteristics

Carcass yield and relative weight of broilers organs as affected by the graded levels of neem oil are presented in Table 4. It can be observed that neem oil had no significant (*P* > 0.05) effect on the carcass yield. Except, for the relative weight of liver which significantly (*P* < 0.05) increased with 25 g of neem oil/kg, the relative weight of organs was not markedly affected by the dietary treatments.

**Table 4:** Carcass yield and relative weight of organs of broiler chickens as affected by graded levels of neem seed oil

Parameters (% of LW)	Controls		Treatments			*P‐*value
	*R*_0_−	*R*_0_+	*R*_15_	*R*_20_	*R*_25_	
Carcass yield	83.86 ± 2.61	85.60 ± 3.71	81.79 ± 6.36	82.90 ± 6.06	82.85 ± 1.80	0.410
Head	2.10 ± 0.14	2.02 ± 0.13	2.02 ± 0.10	2.13 ± 0.20	2.09 ± 0.19	0.360
Feet	3.68 ± 0.49	3.73 ± 0.25	3.54 ± 0.24	3.87 ± 0.47	3.79 ± 0.30	0.351
Heart	0.44 ± 0.05	0.44 ± 0.06	0.42 ± 0.09	0.45 ± 0.06	0.45 ± 0.07	0.878
Liver	2.21 ± 0.28^ab^	2.07 ± 0.19^bc^	1.93 ± 0.23^c^	2.17 ± 0.28^bc^	2.46 ± 0.37^a^	0.003
Pancreas	0.19 ± 0.06	0.22 ± 0.03	0.25 ± 0.06	0.22 ± 0.04	0.22 ± 0.07	0.332
Abdominal fat	0.59 ± 0.28	0.88 ± 0.35	0.88 ± 0.53	0.77 ± 0.31	0.82 ± 0.40	0.449

^a,b,c^Means with different superscript for the same parameters in each row are significantly different (*P* < 0.05). R_0_−: 0 g of neem oil/kg, *R*_0_+: R_0_− + 1 g of Doxycicline/kg, *R*_15_: *R*_0_− + 15 g of neem oil/kg, *R*_20_: *R*_0_− + 20 g neem oil/kg, *R*_25_: *R*_0_− + 25 g of neem oil/kg, *P*: Probability.

### Digestive organs

The development of digestive organs of chickens as affected by the graded levels of neem oil is summarized in Table 5. The intestine density significantly (*P* < 0.05) increased with 20 g of neem oil/kg as compared to antibiotic, while the intestine weight decreased (*P* < 0.05) with antibiotic as compared to the negative control and the treatments containing neem oil.

**Table 5:** Digestive organs of broiler chickens as affected by graded levels of neem seed oil

Digestive organs	Controls		Treatments			*P‐*value
	*R*_0_−	*R*_0_+	*R*_15_	*R*_20_	*R*_25_	
Gizzard (%LW)	1.67 ± 0.26	1.62 ± 0.22	1.51 ± 0.21	1.52 ± 0.27	1.62 ± 0.35	0.597
Intestine weight (%LW)	6.73 ± 1.09^a^	4.42 ± 0.85^b^	6.10 ± 1.24^a^	6.44 ± 0.81^a^	5.96 ± 0.74^a^	0.001
Intestine length (cm)	265.10 ± 26.52	238.00 ± 27.35	255.20 ± 27.14	241.80 ± 26.92	260.40 ± 30.24	0.145
Intestine density (g/cm)	0.52 ± 0.08^ab^	0.41 ± 0.06^c^	0.50 ± 0.07^ab^	0.54 ± 0.09^a^	0.46 ± 0.05^bc^	0.001

^a,b,c^Means with different superscript for the same parameters in each row are significantly different (*P* < 0.05). *R*_0_−: 0 g of neem oil/kg, *R*_0_+: *R*_0_− + 1 g of Doxycicline/kg, *R*_15_: *R*_0_− + 15 g of neem oil/kg, *R*_20_: *R*_0_− + 20 g neem oil/kg, *R*_25_: *R*_0_− + 25 g of neem oil/kg, *P*: Probability.

### Serum biochemical profile of the chicken

Serum content in total protein, total cholesterol, HDL‐cholesterol, AST, ALT and creatinine were not significantly (*P* > 0.05) affected by dietary inclusion of neem oil (Table 6). Urea and LDL‐cholesterol serum content significantly (*P* < 0.05) decreased with 20 g of neem oil/kg feed as compared to the negative and positive control rations, while the triglyceride content significantly (*P* < 0.05) increased with the highest doses of oil (20 and 25 g/kg) as compared to other treatments.

**Table 6:** Biochemicals indices of blood serum in broiler chickens as affected by graded levels of neem seed oil

Parameters	Controls		Treatments			*P‐*value
	*R*_0_^−^	*R*_0_^+^	*R*_15_	*R*_20_	*R*_25_	
Total protein (g/dL)	1.29 ± 0.21	1.92 ± 0.33	1.76 ± 0.16	1.94 ± 0.28	1.79 ± 0.40	0.275
Urea (g/dL)	5.65 ± 1.28^a^	5.04 ± 1.06^a^	3.65 ± 1.42^b^	2.08 ± 1.45^c^	4.34 ± 1.01^ab^	0.000
Creatinine (g/dL)	0.74 ± 0.28	0.41 ± 0.26	0.45 ± 0.46	0.22 ± 0.19	0.74 ± 0.44	0.217
AST (U/L)	203.51 ± 57.29	143.06 ± 62.06	193.52 ± 41.19	270.67 ± 54.93	243.10 ± 56.08	0.809
ALT (U/L)	58.41 ± 11.11	79.19 ± 12.86	58.48 ± 12.43	56.73 ± 12.62	48.56 ± 18.67	0.825
Total cholesterol (g/dL)	89.90 ± 32.73	98.59 ± 25.81	90.78 ± 18.54	81.31 ± 20.77	93.50 ± 22.81	0.719
HDL‐cholesterol (g/dL)	30.40 ± 10.16	33.35 ± 10.31	36.80 ± 21.98	38.55 ± 11.90	38.59 ± 16.69	0.765
LDL‐cholesterol (g/dL)	51.20 ± 38.93^a^	56.97 ± 19.42^a^	42.62 ± 20.33^ab^	17.59 ± 8.63^b^	31.70 ± 14.74^ab^	0.055
Triglycerides (g/dL)	41.46 ± 17.63^b^	41.41 ± 7.23^b^	56.79 ± 28.48^b^	125.82 ± 18.24^a^	115.10 ± 26.07^a^	0.000

^a,b,c^Means with different superscript for the same parameters in each row are significantly different (*P* < 0.05). *R*_0_^−^: 0 g of neem oil/kg, *R*_0_^+^: *R*_0_^−^ + 1 g of Doxycicline/kg, *R*_15_: *R*_0_^−^ + 15 g of neem oil/kg, *R*_20_: *R*_0_^−^ + 20 g neem oil/kg, *R*_25_: *R*_0_^−^ + 25 g of neem oil/kg, *P*: Probability.

## Discussion

The present results showed a non‐significant effect of graded levels of neem oil on feed intake during the entire period of the study. These results are consistent with the report of Landy *et al*. (2011) that indicated no significant effect of dietary inclusion of neem leaves powder at the rate of 7 and 12 g/kg of broiler chickens cumulative feed intake at 42 days. Singh *et al*. (2014), also depicted that there are no significant differences between treatments in the feed intake when adding neem leaves powder to the layer diet at a rate of 1, 2 and 3 g/kg. During the starter phase (days 1 to 21), feeding broilers with 25 g of neem oil/kg of feed resulted in a marked (*P* < 0.05) decrease in feed intake as compared to other treatments. This finding may be explained by the bitter taste of neem oil. During the entire period of the study, the average live body weight and the weight gain decreased with increasing levels of neem oil in the ration. This result contradicted the finding of Manwar *et al*. (2007), who supplemented broilers feed with increasing level of neem leaves powder and reported a significant increase in live body weight when compared with the control group. The present result is not consistent with the report of Onyimonyi *et al*. (2009), which revealed a significant improvement in the live body weight and weight gain when adding neem leaves powder to broiler diet at a rate of 0.5%. The decrease in weight gain could be possibly due to the presence of the various antinutritional factors found in neem seed oil. The highest level of neem oil inclusion (25 g/kg) reduced the growth rate in broilers which could be attributed to the different bio‐active components of neem oil. This is similar to the adverse effect on growth as shown in White Leghorn chicks fed de‐oiled neem seed meal at or above 5% level for 8 weeks (Subbarayudu & Reddy 1975). Salawut *et al*. (1994) also found that the lowest dose of neem oil in the diet of rabbits was better for growth performance than higher levels.

Incorporation of graded levels of neem oil in the ration had no significant (*P* > 0.05) effect on the carcass yield of broilers. These results are in agreement with the findings of Kharde & Soujanya (2014), who reported no significant differences between treatments on carcass parameters when adding neem leaves and garlic powder to broiler diet at a rate of 1.2 g/kg and 0.5 g/kg, respectively. The relative weight of liver significantly (*P* < 0.05) increased in birds fed diets supplemented with 25 g of neem oil/kg. These results are consistent with the study of Durrani *et al*. (2008) that indicated a significant improvement in relative weight when adding 40 mg of neem leaves powder/L of drinking water. In this study, the increase recorded in liver weight can be explained by the intense activity of this organ due to the presence of the antinutritional factors contained in this oil. Indeed, the phenolic compounds and flavonoids contained in this oil have an antioxidant potential with a strong protective activity of liver enzymes. This study revealed that neem oil contained Azadirachtin A (3.46 mg/kg) and Azadirachtin B (0.55 mg/kg) which are the most frequent triterpenoids in the group of flavonols. It is well‐established that azadirachtin is a more potent antioxidant than other antioxidant nutrients such as Vitamin C, vitamin E and *β*‐carotene (Rice‐Evans *et al*. 1995).

Serum content in protein increased with diet supplemented with neem oil/kg as compared to the negative control. Serum protein actually depends on the availability of dietary protein. This suggests that the proteins of neem oil diets were available to the birds. The present result is in line with the findings of Samarth *et al*. (2003), which revealed that incorporation of herbs in the ration increased the serum protein in broilers. The present result suggests that the inclusion of neem oil in the poultry diets did not significantly alter dietary protein utilization and synthesis in the liver or suppress the immune system of the birds. Serum protein is involved in the transport of important body substances and maintenance of normal distribution of water between blood and tissues through osmotic pressure (Polat *et al*. 2011). Low protein is evidence of malnutrition, impaired protein synthesis in the liver or excessive protein catabolism while elevated protein levels may result from dehydration.

Inclusion of 20 g of neem oil/kg of poultry feed induced a significant decrease in LDL‐cholesterol as compared to other treatments. This is consistent with the findings of Ogbuewu *et al*. (2011), Uko *et al*. (2006) and Bonsu *et al*. (2012) who reported the hypocholesterolaemic effects of neem leaf meal in rabbits and broilers, respectively. This study revealed that neem oil contained many compounds such as Quercetin, *β*‐sitosterol (2.70 mg/kg), Stigmastérol (0.50 mg/kg), campestanol (0.01 mg/kg), avenasterol (0.17 mg/kg), oleic acid (0.45%), linoleic (19.78%) linolenic acid (0.45%) and petroselenic acid (0.45%). It is presumed that those compounds either partially or wholly may be responsible for anti‐hyperlipidermic activity of neem oil. The fall in LDL‐cholesterol level of broilers fed neem oil diets probably suggests a general decrease in lipid mobilization; thus indicating that neem oil has indirect inhibitory effects exerted at the levels of HMG‐CoA reductase, a key enzyme in cholesterol biosynthesis.

In the assessment of liver functioning, the activities of AST and ALT in serum were not significantly affected by the graded levels of neem oil. The AST is a cytoplasmic enzyme while ALT is found in both cytoplasmic and mitochondria. According to Bhatti & Dil (2005), alteration in serum enzymes activity under stress conditions occurs due to malfunctioning of liver as degenerating and necrotic cells leak enzymes from cytoplasm. In this study, birds were quite healthy as shown low concentration of liver enzymes. These results corroborate with Ansari *et al*. (2012) reports on the decreasing of the same parameters in birds fed on neem leaf meal as phytogenic feed additive. The non‐hepatoxic nature of neem was reported in the previous study by Haque *et al*. (2006) who recorded unaltered and normal activities of serum AST. In the present study, serum content in AST increased with 20 g of neem oil/kg. Values recorded are range within the reference value (50–350 UI/L) for healthy chickens (Ansari *et al*. 2012). Neem oil is a promising hepatoprotective agent and this protective activity may be due to its antioxidant and normalization of impaired membrane function activity (Mohamed *et al*. 2010).

## Conclusion

The present study revealed that feeding broiler chickens with *Azadirachta indica* seed oil has no detrimental effects on growth performance and hemato‐biochemical parameters. However, the dietary supplementation of this oil up to 20 g/kg may lead to the production of low‐cholesterol chicken meat as demand by health‐conscious consumers. Considering the growing restrictions of antibiotics growth promoters, neem oil could be used as feed additive to mitigate the public concern about bacteria resistance issues as well as antibiotics residues in broiler chickens meat.

## Source of funding

This study is a self funding research.

## Conflicts of interest

The authors declare no conflicts of interests.

## Contribution

MSV and NDK went to the field to carry out the trial and collect the samples. KJR supervised the overall research work, wrote with Mafouo the first draft before being revised and approved by all the authors.

## Ethical Statement

The authors confirm that the ethical policies of the journal, as noted on the journal's author guidelines page, have been adhered to. No ethical approval was required as this is a case report with no original research data. Informed consent was obtained from the client for the publication of this case report.

## References

[vms3162-bib-0001] Ansari S.H. , Islam F. & Sameem M. (2012) Influence of nanotechnology on herbal drugs: A review. Journal Advanced Pharmaceutical Technology of Resources 3, 142–146. 10.4103/2231-4040.101006 PMC3459443 23057000

[vms3162-bib-0002] Bergmeyer B. & Wanlefeld B. (1980) International federation of clinical chemistry scientific committee. Journal of Clinical Chemistry and Clinical Biochemistry 18, 521–534. 7411030

[vms3162-bib-0003] Bhatti B.M. & Dil S. (2005) Effect of vitamin C on immune response in Desi chicken against Newcastle disease. Pakistan Journal of Veterinary Research 2, 48–49.

[vms3162-bib-0004] Bonsu F.R.K. , Kagya‐Agyemang J.K. , Kwenin W.K.J. & Zanu H.K. (2012) Medicinal response of broiler chickens to diets containing neem (*Azadirachta indica*) leaf meal, haematology and meat sensory analysis. World Applied Sciences Journal 19, 800–805.

[vms3162-bib-0005] Durrani F.R. , Sultan A. , Jan M. , Chand N. & Durrani Z. (2008) Immunomodulatory and growth promoting effects of Neem (*Azadirachta indica*) leaves infusion in broiler chicks. Sarhad Journal of Agricultural 24, 655–659.

[vms3162-bib-0006] Haque E. , Mandal I. , Pal S. & Baral R. (2006) Prophylactic dose of neem (*Azadirachta indica*) leaf preparation restricting murine tumor growth is nontoxic, hematostimulatory and immunostimulatory. Immunopharmacology Immunotoxicology 28, 33–50. 16684666 10.1080/08923970600623632

[vms3162-bib-0007] Huyghebaert G. , Ducatelle R. & Van Immerseel F. (2011) An update on alternatives to antimicrobial growth promoters for broilers. Veterinary Journal 18, 182–188. 10.1016/j.tvjl.2010.03.003 20382054

[vms3162-bib-0008] Jahanzeb A. , Sohail H.K. , Ahsan H. & Muhammad Y. (2012) Effect of the levels of *Azadirachta indica* dried leaf meal as phytogenic feed additive on the growth performance and haemato‐biochemical parameters in broiler chicks. Journal of Applied Animal Research 40, 336–345.

[vms3162-bib-0009] Kabir S.M.L. (2009) The role of probiotics in the poultry industry. International Journal Molecular Sciences 10, 3531–3546. 10.3390/ijms10083531 PMC2812824 20111681

[vms3162-bib-0010] Kana J.R. , Mube K.H. , Ngouana T.R. , Tsafong F. , Komguep R. , Yangoue A. & Teguia A. (2017) Effect of dietary mimosa small bell (*Dichostachys glomerata*) fruit supplement as alternative to antibiotic growth promoter for broiler chicken. Journal of World's Poultry Research 7, 27–34.

[vms3162-bib-0011] Kharde K.R. & Soujanya S. (2014) Effect of garlic and neem leaf powder supplementation on growth performance and carcass traits in broilers. Veterinary World 7, 799–802. 10.14202/vetworld.2014.799-802

[vms3162-bib-0012] Koona S. & Budida S. (2011) Antibacterial potential of the extracts of the leaves of *Azadirachta indica* linn. Notulae Scientia Biologicae 3, 65–69.

[vms3162-bib-0013] Landy N. , Ghalamkari G.H. & Toghyani M. (2011) Performance, carcass characteristics, and immunity in broiler chickens fed dietary neem (*Azadirachta indica*) as alternative for an antibiotic growth promoter. Livestock Science 142, 305–309.

[vms3162-bib-0014] Makeri H.K. , Maikai V.A. & Nok J.A. (2007) Effect of topical application of neem seed (*Azadirachta indica*) extract on sheep infested with *Amblyomma variegatum* . African Journal of Biotechnology 6, 2324–2327.

[vms3162-bib-0015] Manwar S.J. , Thirumurugan P. , Konwar D. & Chidanandaiah Karna D.K. (2007) Effect of *Azadirachta indica* leaf powder supplementation on broiler performance. Indian Veterinary Journal 84, 159–162.

[vms3162-bib-0016] Mohamed E.T. , Hisham A.M. & Marwa S.M. (2010) Hepato ameliorative effect of *Azadirachta indica* leaves extract against mercuric chloride environmental pollution. Journal of American Science 6, 735–751.

[vms3162-bib-0017] NRC (National Research Council) (1994). Nutrient Requirements of Poultry: Ninth Revised Edition, 1994. The National Academics Press: Washington, DC 10.17226/2114.

[vms3162-bib-0018] Ogbuewu I.P. , Uchegbu M.C. , Okoli I.C. & Iloeje M.U. (2010) Assessment of blood chemistry, weight gain and linear body measurements of pre‐puberal buck rabbits fed different levels of neem (*Azadirachta indica A. juss*.) leaf meals. Chilean Journal of Agricultural Research 70, 515–520.

[vms3162-bib-0019] Ogbuewu I.P. , Okoro V.M. , Okoli I.C. & Iloeje M.U. (2011) Evaluation of dried leaf meal of an ethnomedicinal plant‐neem on linear growths and reproductive tract morphometry of rabbit does. Electronic Journal of Environmental, Agricultural and Food Chemistry 10, 2153–2159.

[vms3162-bib-0020] Onyimonyi A.E. , Olabode A. & Okeke G.C. (2009) Performance and economic characteristics of broilers fed varying dietary levels of neem leaf meal (*Azadirachta indica*). International Journal of Poultry Science 8, 256–259.

[vms3162-bib-0021] Polat U. , Yesilbag D. & Eren M. (2011) Serum biochemical profile of broiler chickens fed diets containing rosemary and rosemary volatile oil. Journal of Biology and Environment Science 5, 23–30.

[vms3162-bib-0022] Rice‐Evans C.A. , Miller N.J. , Bolwell P.G. , Bramley P.M. & Pridham J.B. (1995) The relative antioxidant activities of plant derived polyphenolic flavonoids. Free Radical Research 22, 375–383. 7633567 10.3109/10715769509145649

[vms3162-bib-0023] Salawut M.B. , Adedeji S.K. & Hassan W.H. (1994) Performance of broilers and rabbits given diet containing full fat neem (*Azadirachta Indica*) seed meal. Animal Production 58, 285–289.

[vms3162-bib-0024] Samarth V.R. , Dakshinkar N.P. , Jagtap D.G. & Bhojne G.R. (2003) Effect of Aswagandha (*Withania somnifera*) on haemato‐biochemical profile of broilers. Indian Journal of Animal Sciences 73, 648–649.

[vms3162-bib-0026] Singh M.K. , Singh S.K. , Sharma R.K. , Singh B. , Kumar S. & Joshi S.K. (2014) Performance and carcass characteristics of guinea fowl fed on dietary Neem (*Azadirachta indica*) leaf powder as a growth promoter. Iranian Journal of Veterinary Research 16, 78–82. PMC4789245 27175156

[vms3162-bib-0027] Steel R.G.D. & Torrie J.H. (1984) Principles and Procedures of Statistics. Inten student edn. McGraw Hill: Tokyo.

[vms3162-bib-0028] Subbarayudu D. , Reddy R . (1975). Utilization of deoiled neem seed cake in chicks ration. Poultry Science symposium. Bhubaneswar. 38p.

[vms3162-bib-0029] Talukdar A.D. , Choudhary M.D. , Chakraborty M. & Dutta B.K. (2010) Phytochemical screening and TLC profiing of plant extracts *Cyathea gigantea* (Wall. Ex. Hook.) Haltt and Cyathea brunoniana Wall. Ex. Hook. (Cl. & Bak.). Journal of Science and Technology: Biological and Environmental Sciences 5, 70–74.

[vms3162-bib-0030] Uko O.J. , Kamalu T.N. , Pindaga U.H. & Rabo J.S. (2006) Studies on toxicity to cockerel chicks of raw full‐fat neem (*Azadirachta indica* Juss) seed kernel. Veterinarski Arhiv 76, 135–144.

[vms3162-bib-0031] Valarmathy K. , Gokulakrishnan M. , Kausar M.S. & Paul K.A. (2010) Study of antimicrobial activity of ethanolic extracts of various plant leaves against selected microbial species. International Journal of Pharmacological Sciences and Research 1, 293–295.

